# The relation between DNA methylation patterns and serum cytokine levels in community-dwelling adults: a preliminary study

**DOI:** 10.1186/s12863-017-0525-3

**Published:** 2017-06-21

**Authors:** Chris P. Verschoor, Lisa M. McEwen, Vikas Kohli, Christina Wolfson, Dawn ME. Bowdish, Parminder Raina, Michael S. Kobor, Cynthia Balion

**Affiliations:** 1 Department of Pathology and Molecular Medicine, McMaster University, 1280 Main St. W, MIP309A, Hamilton, ON Canada; 2 Department of Health Research Methods, Evidence, and Impact, McMaster University, Hamilton, ON Canada; 3 McMaster Institute for Research on Aging, McMaster University, Hamilton, ON Canada; 4 Department of Medical Genetics, Centre for Molecular Medicine and Therapeutics, BC Children’s Hospital Research Institute, University of British Columbia, Vancouver, BC Canada; 5 Canadian Longitudinal Study on Aging, Hamilton, ON Canada; 6 Department of Epidemiology, Biostatistics and Occupational Health, McGill University, Montreal, QC, Canada

**Keywords:** Aging, DNA methylation, Serum cytokine, Epigenetics, Canadian Longitudinal Study on Aging

## Abstract

**Background:**

The levels of circulating cytokines fluctuate with age, acute illness, and chronic disease, and are predictive of mortality; this is also true for patterns of DNA (CpG) methylation. Given that immune cells are particularly sensitive to changes in the concentration of cytokines in their microenvironment, we hypothesized that serum levels of TNF, IL-6, IL-8 and IL-10 would correlate with genome-wide alterations in the DNA methylation levels of blood leukocytes. To test this, we evaluated community-dwelling adults (*n* = 14; 48–78 years old) recruited to a pilot study for the Canadian Longitudinal Study on Aging (CLSA), examining DNA methylation patterns in peripheral blood mononuclear cells using the Illumina HumanMethylation 450 K BeadChip.

**Results:**

We show that, apart from age, serum IL-10 levels exhibited the most substantial association to DNA methylation patterns, followed by TNF, IL-6 and IL-8. Furthermore, while the levels of these cytokines were higher in elderly adults, no associations with epigenetic accelerated aging, derived using the epigenetic clock, were observed.

**Conclusions:**

As a preliminary study with a small sample size, the conclusions drawn from this work must be viewed with caution; however, our observations are encouraging and certainly warrant more suitably powered studies of this relationship.

**Electronic supplementary material:**

The online version of this article (doi:10.1186/s12863-017-0525-3) contains supplementary material, which is available to authorized users.

## Background

There is a wealth of evidence relating the concentrations of circulating cytokines to the severity and outcomes of acute and chronic illness [1]. Some early examples include serum tumour necrosis factor (TNF) with death due following meningococcal meningitis and/or septicaemia [2] and interleukin (IL)-6 with graft rejection following transplantation [3]. A more recent study showed the levels of more than a dozen serum cytokines were indicative of disease subtype for older adults with rheumatoid arthritis [4]. In addition to these pathological forms of stress, other studies have shown that changes in the levels of serum cytokines can also occur in response to physiological (eg. long distance running [5]) and psychological (eg. caregiving [6]) forms of stress as well, which can be reproduced using controlled rodent models [7].

Given the vast array of acute and chronic stressors that individuals experience over their lifespan, it is not surprising that chronological age is accompanied by increases in the levels of circulating cytokine such as IL-6 [6] and TNF [8]. This process is often described within the broader term “inflammaging”, which represents a multi-dimensional chronic inflammatory state that compounds over the trajectory of aging and contributes to premature immunosenescence, morbidity and mortality [9]. Interestingly, these changes in circulating cytokines often parallel alterations in the functionality of circulating blood leukocytes. For example, the production of TNF [10], IL-6 [11], and IL-10 [12] are increased in monocytes from older individuals, and the age-related increase in monocyte IL-6 production in mice can be reversed following the removal of circulating TNF [13]. Although the sub-cellular mechanism mediating the relationship between circulating cytokines and leukocyte function has not been established, an intriguing mechanism for this relationship is the epigenetic regulation of gene expression via DNA methylation [14]. The presence or absence of a methyl group on the 5′ cytosine of cytosine-guanine dinucleotides (CpG) is a critical form of cellular regulation, in particular, the determination of myeloid [15] and lymphoid [16] cell lineage, and the innate immune responses of blood leukocytes [17]. Broad changes to the DNA methylome also occur with age, and are related to epigenetic drift (trajectories that may not be similar amongst individuals) or the epigenetic clock (trajectories that are consistent across the population) [18]. Indeed, both have been reported to be associated with syndromes such as frailty [19], and health deficits such poor physical [20] and cognitive function [20, 21] and coronary heart disease [22] in older adults.

While there is considerable evidence relating DNA methylation patterns with aging, age-related disease and leukocyte function, and aging with circulating cytokine levels and leukocyte function, there are few reports examining the effects of circulating cytokines on DNA methylation patterns, and vice-versa. Results from two independent, targeted studies of the TNF promoter suggest that an age-related loss of DNA methylation increases circulating TNF levels via an increase in transcriptional activity [23, 24]. Another more recent study of nearly 13,000 individuals reported several loci whose DNA methylation levels are significantly associated with changes in the levels of the inflammatory marker, C-reactive protein (CRP) [25]. For the current study, our primary objective was to identify genome-wide DNA methylation patterns that were significantly associated with the levels of circulating cytokines in community-dwelling older adults recruited to a pilot study for the Canadian Longitudinal Study on Aging (CLSA) [26]. We show that the levels of serum TNF, IL-6, IL-8 and IL-10 are significantly associated, with varying degrees, to genome-wide DNA methylation patterns, independent of age and sex. Additionally, as a preliminary study this work validates the sample and data collection, and experimental procedures of the CLSA, thereby supporting larger epigenetic studies in the future.

## Methods

### Participants and serum cytokine analysis

Participants were community-dwelling adults from Hamilton, Ontario and Montreal, Quebec recruited to a pilot study for the Canadian Longitudinal Study on Aging (CLSA) [26]. For the current study, 8 middle-aged (48–55 years old, 4 female) and 6 elderly (72–78 years old, 2 female) individuals were selected from a total of 32 participants recruited for the pilot study. Whole blood was collected between December 2011 and January 2012 and written, informed consent was obtained from all participants. The study protocol and consent procedures were approved by the McMaster Research Ethics Board. Serum levels of IFN-γ, TNF, IL-1β, IL-6, IL-8, IL-10, and IL-12p70 were measured by Milliplex High Sensitivity multiplexed ELISA (Millipore, ON, CA) in November 2012. More than 70% of participants did not have detectable levels of IFN-γ, IL-1β, and IL-12p70 (observed by others [27–30]), so these cytokines were left out of further analyses. Unless stated otherwise, serum cytokine levels were natural-log transformed in order to minimize the effect of extreme values.

### DNA methylation analysis

DNA methylation analysis was performed on cryopreserved PBMCs, prepared between December 2011 and January 2012. Briefly, blood was drawn into CPT vacutainers (BD Biosciences, ON, CA), gently inverted and centrifuged at 1000 xg for 10 mins. Afterwards, the PBMC layer was carefully aspirated, washed twice and resuspended in PBS for storage at −80 °C overnight, and vapour phase liquid nitrogen thereafter. DNA was extracted using the DNeasy Blood Mini Kit (Qiagen, ON, CA) in November 2012. Approximately 750 ng of genomic DNA was then bisulfite converted using the EZ DNA Methylation™ Kit (Zymo Research, CA, USA). Bisulfite converted DNA was then processed using the Infinium HumanMethylation450 BeadChip per manufacturer’s instructions (Illumina, CA, USA).

All processing procedures were performed using the R package ‘minfi’ [31]. Initial probe filtering included removal of the following: control probes designed to interrogate single nucleotide polymorphisms (*n* = 65), those targeted to the X or Y chromosomes (*n* = 11,648), polymorphic (*n* = 20,696) and cross-reactive probes (*n* = 40,590) [32], and those with low detection (detection *p*-value >0.01 on more than two chips; *n* = 434). The final data set included 414,999 probes. Following probe filtering, raw data background correction and dye-bias equalization was performed using Noob [33] and normalization using stratified quantile normalization [31]. Batch effects, namely sentrix ID, were corrected for using the ‘ComBat’ function in the package ‘sva’ [34], and cell mixture effects (relative frequency of monocytes, CD4^+^ and CD8^+^ lymphocytes, NK-cells and B-Cells – see below) were removed from normalized and adjusted beta values using a regression-based approach [35]. Principal component analysis (PCA) was performed using the R package ‘FactoMineR’; the first 11 principal components were selected for analysis following qualitative assessment of the PCA scree plot. DNA methylation age and accelerated aging was calculated using the Horvath DNA methylation age calculator ([36]; https://labs.genetics.ucla.edu/horvath/dnamage/).

### Whole blood immunophenotyping

Peripheral blood leukocyte frequency was measured by multicolour flow cytometry in cryopreserved whole blood in October 2016. For whole blood preparation, blood was drawn into acid-citrate dextrose (ACD) vacutainers (BD Biosciences, ON, CA), gently inverted and mixed 1:1 with 20% DMSO in RPMI (10% DMSO, 50% RPMI final). Aliquots were placed into a CoolCell controlled-rate freezing container (BioCision, CA, USA), stored at −80 °C overnight, and vapour phase liquid nitrogen thereafter. We have previously shown that cryopreserved whole blood is a valid sample type for immunophenotyping [37].

The multicolour stain employed included the following conjugated antibodies: CD45-Alexa Fluor700, CD3-Pacific Blue, and CD14-PE eFluor610 (eBioscience, CA, USA), and CD4-APC Cy7, CD8-Brilliant Violet 510, CD56-PE, NKp46-PE, HLA-DR-PE Cy7, and CD19-FITC (Biolegend, CA, USA). This allowed for the enumeration (relative to CD45^+^ leukocytes) of monocytes (HLA-DR^+^CD14^+^), CD4^+^ and CD8^+^ T-lymphocytes (CD3^+^), natural killer (NK) cells (CD56^+^NKp46^+^), and B-lymphocytes (CD19^+^). Following antibody staining, the samples were fixed and red blood cells lysed with 1× Fix/Lyse Buffer (eBioscience, CA, USA) and washed twice prior to analysis. Samples were analyzed immediately using a Beckman Coulter Gallios flow cytometer (Beckman Coulter, ON, CA), and subsequent gating performed using Kaluza Analysis v1.3 (Beckman Coulter, ON, CA).

### Statistics

All statistics were performed in R version 3.2.3 (R Foundation for Statistical Computing, AUT). Comparison of serum cytokine levels between age groups were performed using the Wilcoxon rank-sum test. Associations with principal component scores and individual CpG methylation loci was performed by multiple linear regression. For regression analyses against principal component scores, age group and sex were assessed together in a single model without serum cytokines, followed by individual models that each included a natural log-transformed cytokine. The methylation levels of individual sites were tested using the R package ‘limma’ [38]. In both cases, M-values were used as opposed to beta methylation values; they represent logit transformed beta values (the ratio of the methylated probe intensity and the overall intensity), and exhibit markedly reduced heteroscedasticity as compared to beta values [39]. For individual CpG sites, adjusted *p*-values were obtained, which represent *p*-values adjusted using Benjamini-Hochberg’s procedure for controlling false discovery rate (FDR). Limma was employed for multivariate analysis as its Bayesian approach for adjusting probe-wise variance has been shown to be superior to traditional linear regression in minimizing false discovery rate [38], and Benjamini-Hochberg’s procedure is the most commonly used approach for minimizing family-wise type I error rate in large-scale “omics” studies. *Post-hoc* simulations indicated that we were powered to detect (β = 80%, α = 1 × 10^−6^) an M-value regression coefficient of 2.0 when comparing age groups and a coefficient of 1.4 for correlations with natural-log transformed cytokines (data not shown).

## Results

**Fig. 1:**
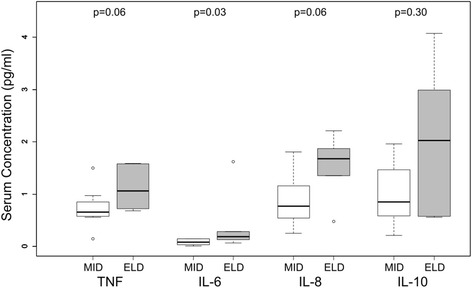
Concentrations of serum cytokines TNF, IL-6, IL-8 and IL-10 in middle-aged (MID; *n* = 8, 48–55 years old) and elderly (ELD; *n* = 6; 72–78 years old) community-dwelling adults. Significance determined by Wilcoxon rank-sum test

High sensitivity, multiplexed bead-ELISAs were performed to measure TNF, IL-6, IL-8 and IL-10 in participant serum (*n* = 14): the median (25th–75th percentile) of TNF was 0.73 (0.65–1.28) pg/ml; IL-6, 0.14 (0.06–0.16) pg/ml; IL-8, 1.16 (0.62–1.73) pg/ml; and IL-10, 1.08 (0.58–1.91) pg/ml. The levels of all cytokines were higher in elderly (*n* = 6; 72–78 years old) as compared to middle-aged (*n* = 8; 48–55 years old) adults, the most significant of which being IL-6 (*p* = 0.03) (Fig. 1).

**Fig. 2:**
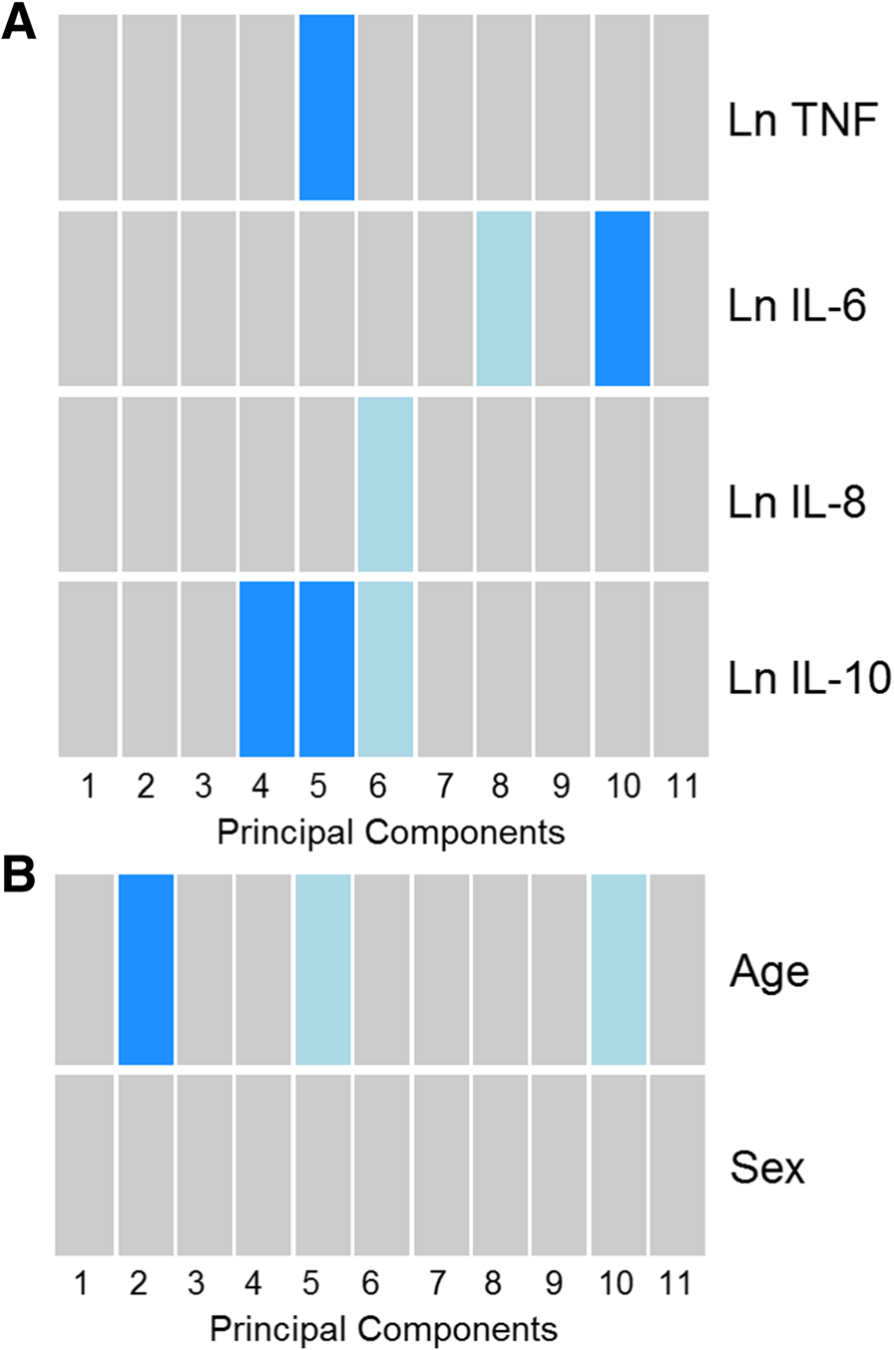
Associations between genome-wide DNA methylation patterns, partitioned using principal component analysis, and age group, sex and serum cytokine levels. Tests were performed by linear regression against the scores for the first 11 principal components. **a**) Natural-log (Ln) transformed serum cytokines were each tested in independent models that also adjusted for age group (middle-aged or elderly) and sex, and **b**) age group and sex were tested together in a single model without serum cytokines. Principal component (PC) 1 represented 22% of the overall DNA methylation variance, PC2 represented 12%, PC3 to PC10 represented between 9.9 and 5.2%, and PC11 represented 1.5%. Significance indicated by colour: dark blue, *p* < 0.05; light blue, *p* < 0.10; grey, *p* > 0.10

In order to assess patterns in genome-wide DNA methylation levels, we partitioned our dataset using principal component analysis (PCA) and used linear regression to test the association between serum cytokine levels and the scores of the first 11 components. Principal component 1 represented 22% of the overall DNA methylation variance, PC2 represented 12%, PC3 to PC10 represented between 9.9 and 5.2%, and PC11 represented 1.5%. Interleukin-10 was associated with principal component (PC) 4 (unadjusted *p* = 0.042) and PC5 (*p* = 0.029), TNF with PC5 (*p* = 0.033), and IL-6 with PC10 (*p* = 0.014) (Fig. 2a). Age group was associated with PC2 (*p* = 0.048; Fig. 2b), while sex did not associate with any PCs.

**Fig. 3:**
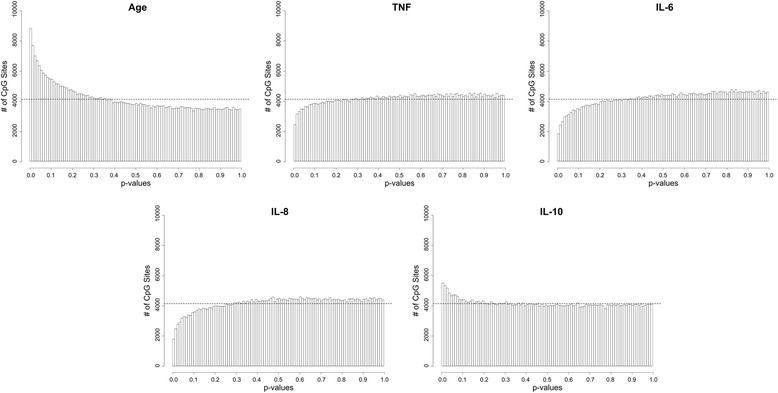
Distribution of significance for each individual DNA methylation site tested against age and serum cytokines levels. Histograms describe the distribution of *p*-values resulting from linear regression tests against DNA methylation M-values, adjusting for age group and sex. Age group was tested as either middle-aged or elderly groups, and serum cytokines were natural-log (Ln) transformed. Dotted lines represent the uniform distribution of *p*-values, in other words, the number of sites expected to be obtained at a given significance level by chance

To determine if individual DNA methylation sites were associated with age or the concentration of serum cytokines, we performed linear regression against the methylation levels of each of the 414,999 probes in our final dataset. This approach yielded few significant observations at an FDR-adjusted *p*-value threshold of 0.05; hence, an unadjusted *p*-value threshold of 1 × 10^−4^ was chosen arbitrarily as a reference point to compare the frequency of significant tests related to each of our variables of interest. As expected, the greatest number of sites were obtained when elderly and middle-aged groups were compared: 129 loci were *p* < 10^−4^ (Fig. 3; Additional file 1: Table S1). Only one loci, cg04267345 (~1kB from the transcriptional start site of Nuclear Factor of Activated T-Cells 4 (NFATC4)), was significantly different between age groups at an FDR-adjusted *p* < 0.05 (unadjusted *p* = 1.08 × 10^−7^); the difference in average methylation frequencies (Δbeta) for this loci was 10.8% (middle-aged = 14.6%, elderly = 3.8%). Regarding serum cytokines, the greatest number of significantly associated sites were obtained for IL-10: 51 loci with *p* < 10^−4^ (zero with an adjusted *p* < 0.05). The effects of the other three cytokines were much lower: TNF, 10 with *p* < 10^−4^; IL-6, 2 with *p* < 10^−4^; and IL-8, 5 with *p* < 10^−4^ (Additional file 1: Table S1). *P*-values for TNF, IL-6 and IL-8 did not follow a uniform distribution, evident by the fewer than expected loci at significance levels below *p* = 0.30 (Fig. 3).

We also measured DNA methylation age, a representative measure of one’s biological age, or the speed at which one is aging. DNA methylation age was highly correlated to chronological age (Spearman’s rho = 0.90) in our participants, however, none of the serum cytokines were associated with DNA methylation age, or age acceleration (ie. rate of aging = DNA methylation age minus chronological age).

## Discussion

In the current study we tested whether variation in the DNA methylation patterns of blood immune cells correlated with serum cytokine levels, namely, TNF, IL-6, IL-8, and IL-10. These molecules can be readily found at detectable levels in older adults (each of these cytokines were identified in >70% of participants in our study), are known to change with age and/or disease state [40–42], and all play instrumental roles in the regulation of inflammation, an important component of several age-related morbidities [9]. We hypothesized that the age-related rise in circulating inflammatory mediators is predicated by a gradual dysregulation of blood immune cell production and secretion of many of those same molecules, a phenomenon that is supported by a number of human ex vivo and rodent experiments from our group and others [10–13].

Comparing serum cytokine levels to DNA methylation patterns, IL-10 exhibited the greatest degree of correlation, followed by TNF, IL-6 and IL-8. As the only cytokine having a primary role in the dampening of inflammatory responses, the observation that its effects were unlike the other three inflammatory cytokines might not be not surprising. Furthermore, IL-10 exhibiting the strongest correlation suggests that circulating leukocytes may exhibit enhanced sensitivity to anti-inflammatory signals, possibly as a protective mechanism against potentially damaging and energy demanding inflammatory responses; this type of mechanism is well described in studies of the intestinal microenvironment [43], as well as in the circulation [44]. As compared to a recent study reporting DNA methylation sites that were significantly correlated with circulating levels of the inflammatory acute-phase protein CRP [25], there are many commonalities to our own results. Of the 58 sites reported by Ligthart and colleagues to be associated to CRP, 25 were identified as being associated (unadjusted *p* < 0.05) with TNF, IL-6, IL-8 and IL-10 in our study. Interestingly, the overwhelming majority of common CpG sites related to inflammatory cytokines in our study (TNF, 7/9 sites; IL-6, 6/6 sites; IL-8, 2/2 sites) exhibited similar relationships (ie. positive or negative correlation) as previously reported for CRP. Regarding the anti-inflammatory cytokine IL-10, of the 5 common sites identified, 4 showed the opposite relationship as to what was previously reported for CRP. These trends suggest that alterations to the leukocyte methylome may occur via inflammatory and anti-inflammatory cues from the microenvironment, as opposed to specific cytokine signaling networks.

Clearly, as a preliminary study with a small sample size, the substantiveness of our findings is limited. The effect of a small sample size is evident in the relatively few CpG loci approaching FDR-adjusted significance as well as the lack of uniformativity of *p*-value distributions for TNF, IL-6 and IL-8. This has been previously observed [45, 46] and may be related to the overestimation of the variance of certain CpG loci due to the small sample size, leading to overly conservative tests and inflated *p*-values [47, 48]. It is worthwhile to note that despite this we were still able to identify at least one DNA methylation site with an FDR adjusted *p*-value <0.05, cg04267345, which showed increased methylation with age. Methylation at a nearby position (~500 bp) has also been shown to increase with age [49], and its closest gene, NFATC4, is prominently involved in the differentiation and development of a number of cell types including neurons, endothelial cells and adipocytes [50].

## Conclusions

In summary, we have provided evidence that the levels of circulating cytokines correlate with the DNA methylation patterns of blood immune cells. Our study provides impetus for future studies on large human aging cohorts such as the Canadian Longitudinal Study on Aging in order to identify correlations between circulating cytokines and DNA methylation patterns with a greater degree of confidence, and importantly, to infer causal relationships via longitudinal analyses.

## Additional file

Additional file 1: Table S1. Differentially methylated position (DMP) analysis of middle-aged and elderly participants. Description: Association of each CpG loci with the variables: age group, Ln IL-10, Ln TNF, Ln IL-6, and Ln IL-8. Analysis performed using limma on M-values, and the difference (log fold-change or regression coefficient) and significance (unadjusted and FDR-adjusted *p*-value, and log-odds difference (B)) presented for all probes with an unadjusted *p*-value <0.05 (XLSX 12493 kb)

## Abbreviations

ACD: Acid-citrate dextrose
CD: Cluster of differentiation
CLSA: Canadian Longitudinal Study on Aging
CpG: Cytosine-guanine dinucleotides
CRP: C-reactive protein
DMP: Differentially methylated position
ELD: Elderly
FDR: False discovery rate
IL: Interleukin
Ln: Natural-log
MID: Middle-aged
NFATC4: Nuclear Factor of Activated T-Cells 4
NK: Natural killer
PCA: Principal component analysis
TNF: Tumour necrosis factor
Δbeta: Difference in average methylation frequencies
